# A Surprising Repurposing of Central Nervous System Drugs against Squamous Cell Carcinoma of the Bladder, UM-UC-5

**DOI:** 10.3390/pharmaceutics16020212

**Published:** 2024-01-31

**Authors:** Maria João Gouveia, Eduarda Ribeiro, Nuno Vale

**Affiliations:** 1 Center for the Study in Animal Science (CECA/ICETA), University of Porto, Rua de D. Manuel II, Apt 55142, 4051-401 Porto, Portugal; mariajoaogouveia@gmail.com; 2 Centre for Parasite Biology and Immunology, Department of Infectious Diseases, National Health Institute Dr. Ricardo Jorge, Rua Alexandre Herculano 321, 4000-055 Porto, Portugal; 3 PerMed Research Group, Center for Health Technology and Services Research (CINTESIS), Rua Doutor Plácido da Costa, 4200-450 Porto, Portugal; eduardaprr@gmail.com; 4 CINTESIS@RISE, Faculty of Medicine, University of Porto, Alameda Professor Hernâni Monteiro, 4200-319 Porto, Portugal; 5 ICBAS—School of Medicine and Biomedical Sciences, University of Porto, Rua Jorge Viterbo Ferreira, 228, 4050-313 Porto, Portugal; 6 Department of Community Medicine, Health Information and Decision (MEDCIDS), Faculty of Medicine, University of Porto, Alameda Professor Hernâni Monteiro, 4200-319 Porto, Portugal

**Keywords:** repurposing drugs, bladder cancer, antineoplastic drugs, central nervous system drugs, UM-UC-5

## Abstract

The potential benefits of drug repurposing have gained attention as an alternative to developing de novo drugs. The potential of using central nervous system (CNS) drugs as anticancer drugs has been explored in several types of human cancers, such as breast and colon cancer, among others. Here, we examine the effect of the CNS drugs sertraline, paroxetine, and chlorpromazine on human squamous carcinoma cells of the bladder (UM-UC-5). After exposing UM-UC-5 cells to increased concentrations of each drug for 48 h, we assessed their metabolic activity using an MTT assay. Based on those results, we calculated cell viability and the half-maximal inhibitory concentration (IC_50_) values. The results suggest that the CNS drugs were effective against UM-UC-5 in the order of potency of sertraline > chlorpromazine > paroxetine. Interestingly, sertraline was more potent than 5-fluorouracil (5-FU), a widely used anticancer drug. This study demonstrated, for the first time, the promising anticancer activity of CNS drugs on human bladder cancer cells in vitro and supports the repurposing of CNS drugs to improve cancer treatment. Nevertheless, further studies are necessary to understand their mechanism of action and in vivo activity.

## 1. Introduction

Bladder cancer is the most common type of cancer that affects the urinary tract system. The latest statistics from the American Cancer Society indicate that approximately 82,290 new cases of bladder cancer were diagnosed in the United States in 2023, resulting in approximately 16,170 deaths [1]. Bladder cancer presents a wide range of histopathological features, with transitional/urothelial carcinomas (T/UCCs) being the most common, followed by squamous cell carcinoma (SCC), adenocarcinoma, and other less common types. However, historically, T/UCCs have received the most research attention, while other malignancies such as SCC remain understudied [2]. Chronic irritation and swelling of the bladder lining can be the main causes that lead to squamous cell carcinoma (SCC). This irritation can cause the normally thin and long transitional cells to transform into flat, scale-like squamous cells over time [3]. SCC of the bladder can be classified into two types: non-bilharzia SCC (NB-SCC) and bilharzia SCC (B-SCC). B-SCC is directly linked to the infection caused by *Schistosoma haematobium*, a blood-dwelling parasite that resides in the vesical plexus of the bladder. After maturation, the parasite produces hundreds of eggs per day, causing chronic irritation in the bladder wall that can eventually lead to B-SCC [4,5,6]. The products released by the eggs can also be implied in the initiation of carcinogenesis associated with the parasite infection [7,8]. B-SCC usually appears earlier in life than NB-SCC and is more prevalent in regions where *Schistosoma haematobium* is endemic, particularly in Sub-Saharan Africa [4,5,6]. Bladder cancer is commonly treated with radical cystectomy, which is known to cause major side effects. The use of chemotherapy as an adjuvant or neoadjuvant, alone or combined with surgery, is not very fully established [4,9]. Therefore, it is crucial to discover new and innovative therapies to efficiently treat these malignancies. 

The discovery and development of a new drug de novo can be very expensive, time-consuming, and often have poor success rates in reaching Phase I clinical trials [10]. Consequently, drug repurposing has gained attention as it provides a faster and more cost-effective way to access novel therapies [11,12]. This strategy involves the use of drugs that have already been approved by the Food and Drug Administration (FDA) or similar entities to evaluate if they have a clinical impact on other diseases beyond their original indication. One of the advantages of drug repurposing is that these drugs already have a well-defined toxicological and pharmacokinetic profile, which increases the probability of proceeding faster to advanced phases in clinical trials. Clinical trials remain important in this strategy since it is indispensable to evaluate potential novel side effects that may not be observed in its primary indication [11,12,13,14]. 

**Figure 1:**
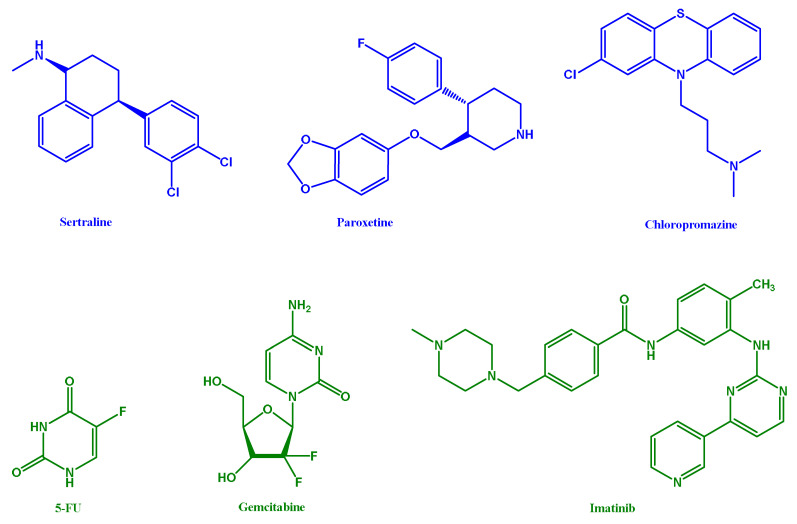
Chemical structures of drugs evaluated in this study against SCC of the bladder. CNS drugs sertraline (STL), paroxetine (PXT), and chlorpromazine (CPM) are depicted in blue. Antineoplastic drugs 5-fluorouracil (5-FU), gemcitabine (GCB), and imatinib mesylate (IMT) are highlighted in green.

Several studies have indicated that central nervous system (CNS) drugs may be effective in suppressing the growth of cancer cell lines, e.g., colon, breast, and others [15,16,17,18,19,20,21,22,23]. Based on this knowledge, we hypothesized whether CNS drugs exhibit anticancer activity against SCC of the bladder when compared to antineoplastic drugs (depicted in Figure 1). 

CNS drugs can be classified into several categories, including antidepressants and antipsychotics, which were the types of drugs used in the study. Sertraline (STL) and paroxetine (PXT) are commonly used as antidepressants and act by selectively inhibiting the reuptake of serotonin, which increases the synaptic concentration of serotonin in the CNS, relieving symptoms of depression and anxiety-related disorders [24,25,26]. Chlorpromazine (CPM), on the other hand, has been used for over 70 years as an antipsychotic medication and is still one of the most commonly prescribed drugs for treating schizophrenia [27]. It acts as an antagonist of various postsynaptic receptors, such as dopaminergic, serotonergic, histaminergic, alpha1/alpha2, and muscarinic M1/M2 receptors [28]. Curiously, these CNS drugs have previously demonstrated anthelmintic activity against developmental stages of *Schistosoma*, inducing changes in parasite morphology and even death [29]. In the case of B-SCC, this is particularly relevant since they may not only eliminate the parasite but also halt the progression of SCC of the bladder associated with *Schistosoma haematobium* infection.

Herein, we also evaluated antineoplastic drugs, namely 5-fluorouracil (5-FU), gemcitabine (GCB), and imatinib mesylate (ITM) (Figure 1). 5-FU is considered an antimetabolite that disrupts DNA synthesis, thus preventing the division of cancer cells. It is commonly used to treat colon and basal cell carcinomas [30]. GCB is an antimetabolite drug that is used to treat various types of cancer, including bladder cancer. Similar to 5-FU, GCB promotes apoptosis of malignant cells undergoing DNA synthesis [30]. ITM is a kinase inhibitor that blocks the action of the abnormal protein that signals cancer cells to multiply. This drug is predominantly used to treat patients with chronic myeloid leukemia and malignant gastrointestinal stromal tumors [31]. 

To our knowledge, this is the first study to evaluate the cytotoxic effects of sertraline, paroxetine, and chlorpromazine against SCC of the bladder. It is interesting to note that these drugs have demonstrated greater potency than ITM, which is an antineoplastic drug. Moreover, their anticancer activity is similar to that of 5-FU, which indicates that they have the potential to be effective anticancer agents. While more research is needed in this area, the potential benefits of using CNS drugs for the treatment of SCC of the bladder are certainly worth exploring further.

## 2. Materials and Methods

### 2.1. Prediction of Potential Targets

To identify potential drug targets, SwissTargetPrediction, available at http://www.wisstargetprediction.ch (accessed on 21 November 2023), serves as a widely used database for forecasting compound targets. In our study, we employed it to predict the potential targets of 5-FU, gemcitabine, imatinib, sertraline, paroxetine, and chlorpromazine. The Human Protein Atlas database, at www.proteinatlas.org (accessed on 21 November 2023), was used to collect information on potential targets associated with the primary active chemical components of the six drugs used in this study.

### 2.2. Cell Material and Drugs

Dulbecco’s modified Eagle’s medium (DMEM), fetal bovine serum (FBS), and penicillin–streptomycin solution were purchased from PAN Biotech (Aidenbach, Germany). Trypsin (TrypLE^TM^ Express 1X, cat. no. 12605-010) was purchased from Gibco (Thermo Fisher Scientific Inc, Waltham, MA, USA), and phosphate-buffer solution (PBS) from Corning (New York, NY, USA). 

Sertraline, paroxetine, chlorpromazine, 5-FU, gemcitabine, and Thiazolyl Blue Tetrazolium Bromide were obtained from Millipore Sigma (Merck KGaA, Darmstadt, Germany) and dimethyl sulfoxide (DMSO, cat. no. D4540) was obtained from Sigma-Aldrich (Merck KGaA, Darmstadt, Germany). Imatinib mesylate (cat. no. 5906) was purchased from Tocris Bioscience (Bristol, UK).

### 2.3. Cell Line and Cell Culture

Human squamous cell carcinoma of the bladder UM-UC-5 (cat. no. 08090502) was obtained from the European Collection of Authenticated Cell Cultures (ECACC, United Kingdom). Cells were maintained at 37 °C and 5% CO_2_ in DMEM medium. This medium was supplemented with 10% FBS, 100 U/mL penicillin G, and 100 µg/mL streptomycin in T25 cm^2^ flasks. The medium was renewed every 2–3 days and split when 70–80% confluence was reached. For this procedure, cells were trypsinized for 7 min at 37 °C and subcultured in the same medium. 

UM-UC-5 cells were seeded in a 96-well plate with 25,000 cells per well and allowed to adhere overnight before drug exposure. The cell culture medium was then replaced with drug-containing media and incubated for 48 h. After that, the cytotoxic effect of each drug on cell viability was determined using an MTT assay [16,17,18]. 

### 2.4. Cell Viability Assay

To evaluate the antitumor effect of CNS drugs (sertraline, paroxetine, chlorpromazine) and antineoplastic drugs (imatinib, 5-FU, and gemcitabine) in UM-UC-5 cells, the MTT assay was conducted. Briefly, after 48 h of drug exposure, the cell medium was removed, and 100 µL/well of MTT solution (0.5 mg/mL in PBS) was added. The cells were then incubated for 3 h at 37 ºC, protected from light. At the end of this period, the MTT solution was removed, and DMSO (100 µL/well) was added to solubilize the formazan crystals. Absorbance was measured at 595 nm in an automated microplate reader (FluorStar Omega, Biotek Instruments Inc., Winooski, VT, USA). The experiments were conducted in triplicate and repeated at least twice [16,17,18].

### 2.5. Cytotoxicity Assay

To determine the half-maximal inhibitory concentration (IC_50_) value for each drug in UM-UC-5, the drugs were first dissolved in 100% DMSO with ranging concentrations of 100-0.1 mM and then diluted in cell culture media to concentrations between 0.01 and 100 µM for the treatment of cells with each CNS drug. Antineoplastic drugs (IMT, 5-FU, GCB) were evaluated in concentrations of 0.01 to 10 µM. The cells treated with vehicle (0.1% DMSO) were used as the control [16,17,18].

### 2.6. Cell Morphology Visualization

After the 48 h drug exposure, we used a digital inverted microscope (EVOS M5000 Imaging System, Thermo Fisher Scientific Inc, Waltham, MA, USA) to visualize and capture the morphological characteristics of UM-UC-5 cells.

### 2.7. Data Analysis

To obtain the cell viability graphs and concentration–response curve with non-linear regression analysis, we used GraphPad Prism 6 (GraphPad Software Inc., San Diego, CA, USA). We represented the cell viability as the mean ± SEM for n experiments performed. Statistical analysis was performed with a one-way ANOVA test by Dunnett’s multiple comparisons between control vs. treatment groups and considered statistically significant at *p* values < 0.05. For IC_50_ values, the cell viability of treated groups was normalized to the viability of control cells, and cell viability fractions were plotted vs. drug concentration in the logarithmic scale.

## 3. Results

### 3.1. Targets of the Drugs

Using the molecular structure of 5-FU, gemcitabibe, imatibin, sertraline, paroxetine, and chlorpromazine, as shown in Figure 1, the online tool SwissTargetPrediction was used to predict 100 potential targets for each drug (Table 1). 

According to the literature, G protein-coupled receptors (GPCRs) are a large family of cell surface receptors that play a crucial role in transmitting signals from the external environment to the interior of the cell. They are involved in various physiological processes, and their deregulation has been implicated in many diseases, including cancer [32]. The involvement of family A GPCRs in cancer can manifest itself in different ways. These receptors can be over-expressed in cancer cells, leading to increased signaling and promoting cell proliferation, survival, and migration. Alternatively, mutations in GPCR genes can result in constitutive activation, bypassing normal regulatory mechanisms [33].

After identifying the drug-related targets in SwissTargetPrediction, the targets with the highest probability of binding to drugs were searched for in the Human Protein Atlas database. Considering the crucial role of in silico studies in precisely examining study subjects under controlled conditions, their fundamental significance in identifying potential drugs targeting tumor markers becomes apparent. 

**Figure 2:**
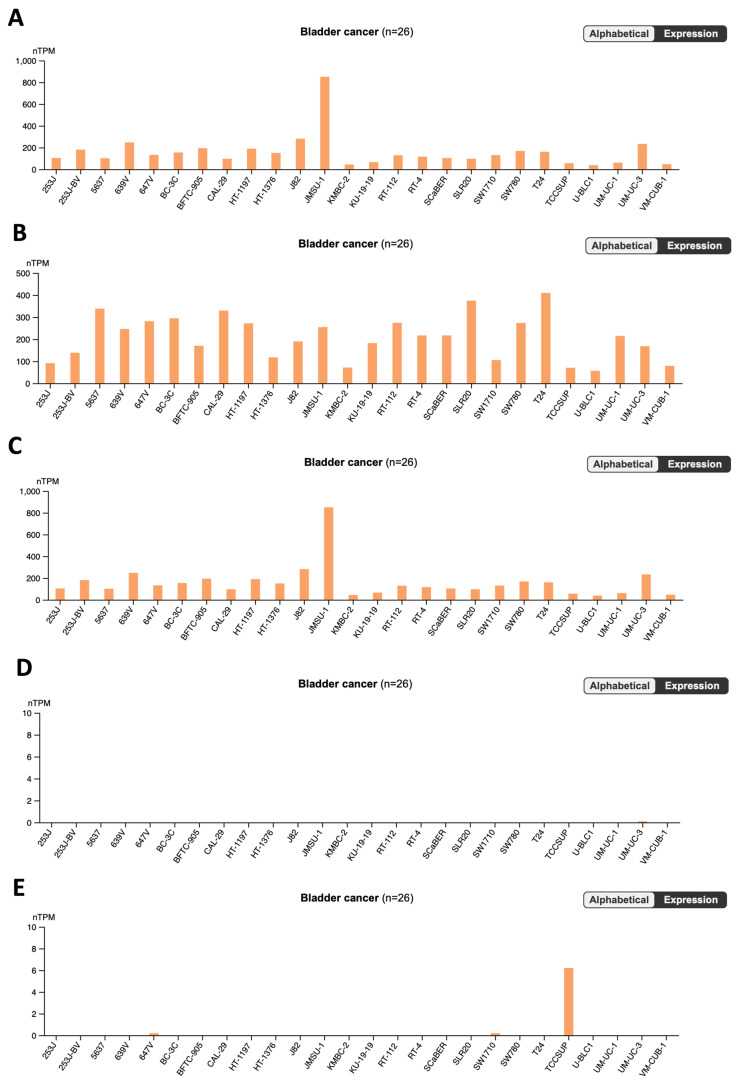
Cell line RNA expression data of reference drugs: (**A**) TYMS gene, (**B**) TK1 gene, (**C**) CDA gene, (**D**) HIPK4 gene, and (**E**) SLC22A2 gene from Human Protein Atlas database.

**Figure 3:**
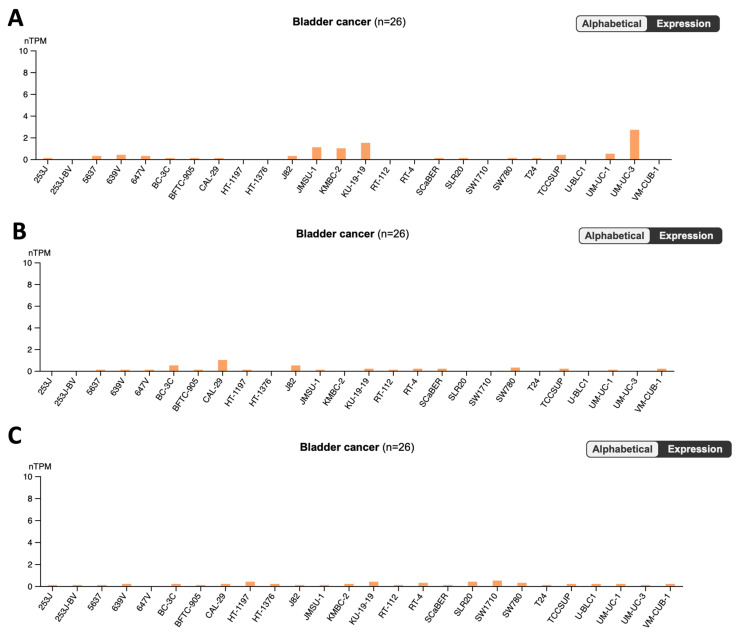
Cell line RNA expression data of CNS drugs: (**A**) CHRM4 gene, (**B**) HTR2B gene, and (**C**) CHRM5 gene from Human Protein Atlas database.

Figure 2 and Figure 3 shows the graphs generated from the Human Protein Atlas platform in the search for the genes most likely to bind to the drugs in this study in bladder cancer cell lines.

The results linked to the reference drugs highlight a significant contrast in cytotoxicity between the experimental IMT drug and the comparator medications, GCB and 5-FU, in UM-UC-5 cells. Notably, the anticipated lower cytotoxicity of IMT is attributed to the observed lower expression levels of its primary targets, HIPK4 and SLC22A2, in bladder cancer compared to the targets of GCB and 5-FU.

As a result of this comprehensive research endeavor, a trove of valuable discoveries has emerged, with particular emphasis on the identification of STL, PXT, and CMP ligands. The pivotal conclusion drawn from this investigation underscores the significant expression of the respective targets associated with these ligands in bladder cancer. This discerning finding not only highlights the potential therapeutic relevance of STL, PXT, and CMP in the context of bladder cancer treatment but also accentuates the prospect of targeted interventions leveraging these ligands to address the specific molecular landscape of the disease. The recognition of substantial target expression levels amplifies the rationale for further exploration and development of these compounds as promising candidates for novel therapeutic strategies in the realm of bladder cancer research.

### 3.2. Central Nervous System Drugs Present Promising Antineoplastic Activity against Squamous Cell Carcinoma of the Bladder Cells

**Figure 4:**
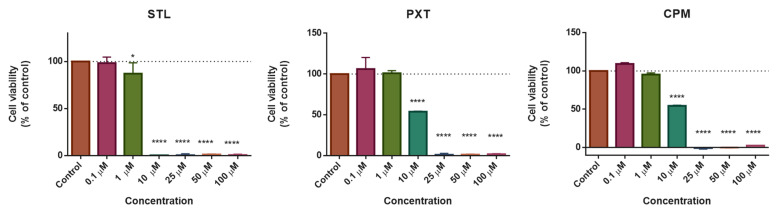
Viability of UM-UC-5 bladder cancer cells incubated with different CNS drugs. Cells were seeded in 96-well plates and treated with increaing concentrations from 0.1 to 100 µM. Cells treated with vehicle (0.1% DMSO) were used as control. Cell viability was evaluated using an MTT assay after 48 h of drug exposure. Each bar represents the mean ± SEM relative to the control cells. * Statistically significant vs. control at *p* < 0.05. **** Statistically significant vs. control at *p* < 0.0001.

The cytotoxic potential of CNS drugs (STL, PXT, and CPM) was evaluated in human squamous cell carcinoma of the bladder cell line UM-UC-5. Firstly, we treated UM-UC-5 cells with varying concentrations of STL, PXT, and CPM, ranging from 0.1 to 100 µM. Cell viability was calculated using the data obtained in the MTT assay that measures mitochondrial activity [34]. The results demonstrated that treatment with CNS drugs resulted in concentration-dependent growth inhibition (Figure 4). At lower concentrations (0.1 and 1 µM), the reduction in cell viability was not statistically significant in comparison to the control, except for STL. STL was the most cytotoxic CNS drug against UM-UC-5. When treated with PXT and CPM at a concentration of 10 µM, UM-UC-5 viability was significantly decreased, leading to the death of nearly 50% of cells. The percentage of cell death was increased to over 95% when CNS drugs were evaluated at concentrations exceeding 25 µM. The three CNS drugs (STL, PXT, and CPM) exhibited cytotoxic potential, suggesting that they may have promising antitumor activity against these cancer cells. 

**Figure 5:**
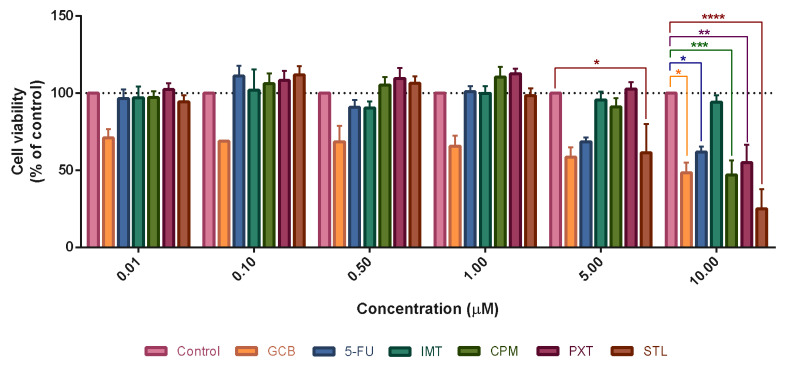
Viability of UM-UC-5 bladder cancer cells incubated with different CNS drugs (STL, PXT, and CPM) and antineoplastic drugs (IMT, 5-FU, and GCT) using MTT assay followed by 48 h of drug exposure. Cells were seeded in 96-well plates and treated with increasing concentrations from 0.1 to 100 µM. Cells treated with vehicle (0.1% DMSO) were used as control. Each bar represents the mean ± SEM relative to the control cells. * Statistically significant vs. control at *p* < 0.05. ** Statistically significant vs. control at *p* < 0.01. *** Statistically significant vs. control at *p* < 0.001. **** Statistically significant vs. control at *p* < 0.0001.

Since CNS drugs had antitumor activity at higher concentrations, we performed a secondary screening using a range of concentrations from 0.01 to 10 µM. In this screening, we also included three antineoplastic drugs (IMT, GCB, and 5-FU) to compare their potency with CNS drugs. Cell viability results demonstrated that all CNS drugs were cytotoxic to UM-UC-5 cells at a concentration of 10 µM (Figure 5). At this concentration, STL caused a significant reduction in cell viability to approximately 25%, followed by CPM (~50%) and PXT (~60%). Moreover, STL decreased cell viability at 5 µM, rendering it the most active of CNS drugs in UM-UC-5 cells as observed in the primary screening. 

Concerning antineoplastic drugs, UM-UC-5 cells were found to be susceptible to GCB and 5-FU; however, IMT did not affect cell viability (Figure 5). While GCB decreased cell viability at all concentrations, 5-FU was active at higher concentrations (5 and 10 µM). Nonetheless, both drugs led to a statistically significant reduction in cell viability at 10 µM. Remarkably, when comparing the effect of CNS drugs with antineoplastic drugs at this concentration, STL and CPM were found to be more or similarly effective than antineoplastic drugs. STL was even more active than GCB, a reference drug for bladder cancer treatment, as depicted in Figure 5.

**Figure 6:**
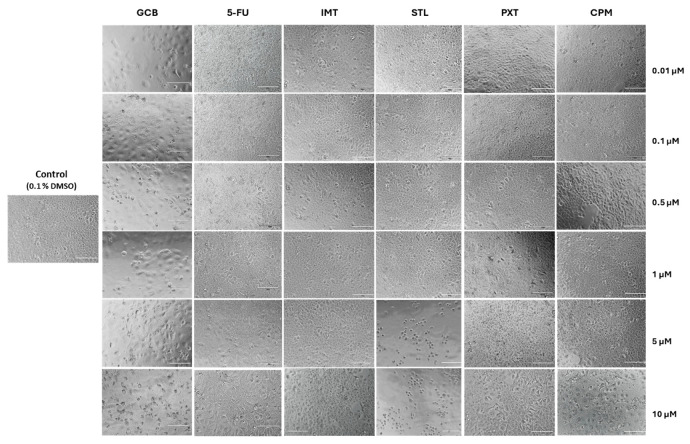
Morphology of UM-UC-5 cells treated with increased concentrations of CNS drugs and antineoplastic drugs for 48 h. Micrographs were captured by a digital inverted microscope EVOS M5000 Imaging System (magnification 20×). The scale bar is 150 µm.

The morphology of cells treated with different drugs and controls after 48 h of drug exposure is shown in Figure 6. In general, the treated cells displayed a different morphology compared to the controls. With an increase in concentrations, cells treated with STL became rounder and more granulated while those treated with PXT and CPM were elongated. Additionally, treatment with CPM led to a large number of cell debris in wells, suggesting a potential increase in cell activity. The antineoplastic drugs 5-FU and GCB also altered cell morphology. 5-FU rendered cells more elongated while GCB induced a significant amount of cell lysis and debris. As expected, cells treated with IMT were morphologically similar to controls. At lower concentrations, a comparison of treated cells and controls demonstrated no significant morphological alterations. Thus, the observations resulting from morphological visualization are in line with the data obtained by MTT. 

Table 2 depicts the IC_50_ values calculated for each drug obtained by plotting the cell viability data obtained through the MTT assay following the generation of curve–dose response. These values serve as indicators of drug efficacy and represent the concentration that reduces cell viability by 50% between the top and bottom plateaus of the normalized dose–response curve obtained through non-linear regression analysis.

Predictably, GCB demonstrated the highest antitumor potency with an IC_50_ of 11.55 nM. Notably, all CNS drugs tested in UM-UC-5 were found to be cytotoxic. Upon comparing the IC_50_ values, it was observed that sertraline > chlorpromazine > paroxetine in terms of potency. It is interesting to note that STL was found to be 3-fold more potent than the antineoplastic drug 5-FU. The CNS drugs tested had IC_50_ values below 10 µM, which supports the idea that they could be promising candidates for drug repurposing in the chemotherapy of SCC of the bladder.

## 4. Discussion

Cancer treatment has long relied on chemotherapy as the primary method of treatment. However, the process of developing a new de novo drug is not only costly but also time-consuming, with no guarantee of success. As a result, alternative strategies have been gaining attention (e.g., use of epigenetic drugs) [35,36]. One such strategy is drug repurposing, which involves using drugs already approved for humans for treating a different disease. This approach is attractive because the pharmacological and toxicological properties of these drugs are already described, making it easier to introduce them for new indications [37]. 

Over the past few years, studies have assessed the antitumor effects of several CNS drugs on different cancer cell lines [15,17,18]. Here, for the first time, we hypothesized that CNS drugs (sertraline, paroxetine, and chlorpromazine) show anticancer activity on UM-UC-5 human bladder cancer cells. The in silico results shows that the protein most likely to be targeted by these molecules was the Family A G protein-coupled receptor for CNS drugs, transferase for 5-FU and GCB, and kinase/electrochemical transporters for IMT. Looking at the outcomes associated with the reference drugs, it becomes evident that the IMT drug is anticipated to exhibit lower cytotoxicity in UM-UC-5 cells when contrasted with GCB and 5-FU. This projection stems from the observation that the primary targets for IMT (HIPK4 and SLC22A2) are not expressed as significantly in bladder cancer compared to the targets of GCB and 5-FU. This variation in target expression levels suggests a potentially less cytotoxic impact of IMT on UM-UC-5 cells, positioning it as a weak candidate with an insufficient mechanism of action in the context of bladder cancer treatment. To test this hypothesis, cells were incubated with increased concentrations of each drug, and metabolic activity was assessed using an MTT assay to determine cell viability. The in vitro results show that CNS drugs, especially STL, have promising anticancer activity against UM-UC-5 human bladder cancer cells. All CNS drugs evaluated decreased the cell viability of UM-UC-5 in a concentration-dependent manner. Interestingly, STL was more active than the widely used antineoplastic drug 5-FU. This suggests the potential of repurposing CNS drugs for cancer treatment, either alone or in combination with reference drugs for bladder cancer. The combination of different CNS drugs with antineoplastic drugs has shown promising antitumor activity in colon and breast cancer treatment [15,17,18]. It is hypothesized that a similar outcome would be observed using a combination of STL, PXT, and CPM with reference drugs for bladder cancer. This could diminish the side effects and improve the anticancer activity of chemotherapy.

The anticancer activity of sertraline, paroxetine, and chlorpromazine on different cancer cell lines was previously described. Sertraline was found to be effective against several human cancer cell lines ([15,38,39,40,41,42,43] reviewed in [19]) (Table 3). Through the comparison of the values obtained for those cells with UM-UC-5 (Table 2), it is possible to observe that the latter are more sensitive to STL. The consonance with these results suggests that STL presents a broad antitumor activity. This may be related to the hypothesis that STL reduces tumor growth by inhibiting the PI3K/Akt/mTOR signaling pathway, which is a growth pathway that is active in multiple cancers [39]. It was also suggested that it could disrupt redox balance, leading to an increase in the expression of autophagy markers and apoptosis [38,41]. The mechanism of action on UM-UC-5 is probably similar to that described for other cancer cells.

Similarly, the potential anticancer activity of PXT was also evaluated in several cancer cell lines, namely colon, liver, and osteosarcoma [43,44,45,47,48,49]. The findings showed that PXT significantly decreased the viability of liver and colon cancer cells (Table 2) [44,45]. Here, PXT was also demonstrated to be cytotoxic against the UM-UC-5 bladder cancer cell line. Interestingly, the IC_50_ value of UM-UC-5 was similar to that of HT29 and HepG2, despite being derived from different organs (i.e., colon, liver, and bladder) (Table 2 and Table 3). This suggests that these cancer cells share some common features that make them sensitive to PXT. Previous studies have suggested that PXT inhibits the activation of major tyrosine kinases, MET and ERBB3, which leads to the suppression of AKT, ERK, and p38 activation, and the induction of the JNK and caspase-3 pathways [44]. However, it was observed that IMT, a tyrosine kinase inhibitor, did not affect the cell viability of UM-UC-5. IMT is a competitive inhibitor of BCR-ABL, KIT, and platelet-derived growth factor receptors (PDGF-R) [50]. It is likely that these tyrosine kinases might not be present in UM-UC-5 bladder cancer cells. In addition to inducing apoptosis through extracellular Ca^2+^ and p38 MAPK-dependent ROS generation in human breast cancer MCF-7 cells [48], PXT has also been found to influence Ca^2+^ movement in OC2 human oral cancer cells and osteosarcoma [49]. Interestingly, a similar effect was observed using STL in OC2 human oral cancer by Chien et al. [51]. 

On UM-UC-5 bladder cancer cells, CPM has been found to have anticancer activity similar to that of 5-FU. Both drugs showed similar IC50 values (Table 2), indicating that CPM has promising potential as an anticancer agent. Interestingly, the cellular morphology of cells treated with CPM showed more alterations than those treated with 5-FU (as shown in Figure 6). The potential of CPM as an anticancer agent was first suggested more than three decades ago, and since then, its cytotoxic effect on multiple cancer cell lines has been studied extensively [46,52,53,54,55,56,57,58]. Studies have shown that CPM decreases cell viability in an apoptosis-independent manner on different glioblastoma cell lines, with an IC_50_ value ranging between 21.6 and 10.3 µM, depending on the cell line (Table 3). Moreover, the use of CPM in combination with a reference drug, temozolomide, has shown a synergistic effect [46]. These results have led to the launch of a Phase II clinical trial on GBM patients, where they will be treated with a combination of CPM and temozolomide (EudraCT # 2019-001988-75; ClinicalTrials.gov Identifier: NCT04224441). The fact that CMP affects UM-UC-5 cell viability, achieving an IC_50_ at least 2-fold lower than that described on glioblastoma cell lines, reinforces the pursuit of repurposed CNS drugs used alone or in combination for cancer treatment. 

It is worth noting that STL, PXT, and CPM have demonstrated anthelmintic activity against the parasite *Schistosoma*, effectively killing most of the parasites. On the other hand, CPM did not show any anthelmintic activity [59]. The anthelmintic activity of these drugs is particularly relevant because *Schistosoma haematobium* is considered a carcinogenic parasite, and its infection is directly associated with the development of squamous cell carcinoma of the bladder [60]. Given the anticancer activity observed here against UM-UC-5 and the anthelmintic activity against parasites, these CNS drugs have a dual activity that can help eliminate the parasite and counteract the development/progression of bladder cancer. 

This study has limitations as we did not evaluate cytotoxicity in noncancerous bladder cells or use other similar tumor models. Yet, as the drugs we tested are approved for human use, the risk of cytotoxicity is low. To summarize, the CNS drugs evaluated here exhibited promising anticancer activity against UM-UC-5 bladder cancer cells. While these in vitro results are encouraging, further research is needed to fully comprehend the anticancer mechanism of action of these drugs. To address another study’s limitations, we will use animal models to evaluate both in vivo anticancer activity and potential neurological side effects in the future. Nevertheless, this study highlights for the first time the potential of CNS drugs as repurposed drugs for the treatment of squamous cell carcinoma of the bladder that can be developed with a parasitic infection. 

## Figures and Tables

**Table 1:** Predicted targets of 5-FU, GCB, IMT, STR, PTX, and CMP from SwissTargetPrediction database.

Drugs	Target Class	Target (Common Name)	Role of Target
5-Fluorouracil	Transferase	TYMS	Biosynthesis of thymidylate
Gemcitabine	Transferase Enzyme	TK1 CDA	Recycling and regenerating thymidine for DNA synthesis DNA and RNA synthesis
Imatinib	Kinase Electrochemical transporter	HIPK4 SLC22A2	Regulates phosphorylation Mediates the transport of a variety of organic cations
Sertraline	Family A G protein-coupled receptor	CHRM4 HTR2B	Adenylyl cyclase inhibition Inhibition of serotonin
Paroxetine		CHRM4 CHRM5	Adenylyl cyclase inhibition Adenylate cyclase inhibition
Chlorpromazine		CHRM4 HTR2B	Adenylyl cyclase inhibition Inhibition of serotonin

TYMS, thymidylate synthase; TK1, thymidine kinase 1; CDA, cytidine deaminase; HIPK4, homeodomain-interacting protein kinase 4; SLC22A2A, solute carrier family 22 member 2; CHRM4, cholinergic receptor muscarinic 4; HTR2B, 5-hydroxytryptamine receptor 2B; CHRM5, cholinergic receptor muscarinic 5.

**Table 2:** IC_50_ values obtained for CNS and antineoplastic drugs in UM-UC-5 in this project.

Class of Drug	Drug	IC_50_ (µM)
Central nervous system	Sertraline	1.90
	Paroxetine	8.45
	Chlorpromazine	5.03
Antineoplastic	5-FU	4.10
	Gemcitabine	0.0116
	Imatinib	>10.00

**Table 3:** Anticancer activity of CNS drugs on several cell lines.

Drug	Type of Cancer	Cell Line	IC_50_ (µM)	References
Sertraline	Breast	MCF-7	2.22	[38]
	Gastric	SGC-7901/DDP	18.73	[39,41]
	Prostate	PC3	N.D.	[40]
		DU145		[40]
		LNCaP		[40]
		PSCS ^1^		[40]
	Colon	HT-29	2.45	[15]
			14.7	[42]
		LS1034	13.1	[42]
	Lung	A549	11.10	[43]
		H522	10.50	
		PC9/R	9.60	
		H1975	9.40	
Paroxetine	Colon	HCT116	13.50	[44]
		HT-29	7.01	
	Liver	HepG2	7.30	[45]
Chlorpromazine	Glioblastoma	T98G	10.3	[46]
		U-87 MG	10.4	
		U-251 MG	10.6	
		TS#1 ^2^	21.6	
		TS#83 ^2^	18.4	
		TS#163 ^2^	15.4	

^1^ PSCS: prostate stem cancer cells; ^2^ patient-derived cell lines from surgical samples. N.D.: not determined.

## Data Availability

The data presented in this study are contained within the manuscript.
